# Discovery of Novel *Plasmodium falciparum* Pre-Erythrocytic Antigens for Vaccine Development

**DOI:** 10.1371/journal.pone.0136109

**Published:** 2015-08-20

**Authors:** Joao C. Aguiar, Jessica Bolton, Joyce Wanga, John B. Sacci, Hideyuki Iriko, Julie K. Mazeika, Eun-Taek Han, Keith Limbach, Noelle B. Patterson, Martha Sedegah, Ann-Marie Cruz, Takafumi Tsuboi, Stephen L. Hoffman, Daniel Carucci, Michael R. Hollingdale, Eileen D. Villasante, Thomas L. Richie

**Affiliations:** 1 Malaria Department, Naval Medical Research Center, Silver Spring, MD 20910, United States of America; 2 Camris International, Bethesda, MD 20814, United States of America; 3 The Henry M. Jackson Foundation for the Advancement of Military Medicine, Inc., Bethesda, MD 20817, United States of America; 4 Technical Resources International, Inc., Bethesda, MD 20817, United States of America; 5 Department of Microbiology and Immunology, The University of Maryland School of Medicine, Baltimore, MD 21201, United States of America; 6 Department of International Health, Kobe University Graduate School of Health Science, Kobe 654-0142, Japan; 7 EMD Millipore Corporation, North Andover, MA 01845, United States of America; 8 Department of Medical Environmental Biology and Tropical Medicine, School of Medicine, Kangwon National University, Chuncheon, Gangwon-do 200-701, Republic of Korea; 9 PATH Malaria Vaccine Initiative, Washington, DC 20001, United States of America; 10 Proteo-Science Center, Ehime University, Matsuyama, Ehime 790-8577, Japan; INSERM, FRANCE

## Abstract

**Background:**

Nearly 100% protection against malaria infection can be achieved in humans by immunization with *P*. *falciparum* radiation-attenuated sporozoites (RAS). Although it is thought that protection is mediated by T cell and antibody responses, only a few of the many pre-erythrocytic (sporozoite and liver stage) antigens that are targeted by these responses have been identified.

**Methodology:**

Twenty seven *P*. *falciparum* pre-erythrocytic antigens were selected using bioinformatics analysis and expression databases and were expressed in a wheat germ cell-free protein expression system. Recombinant proteins were recognized by plasma from RAS-immunized subjects, and 21 induced detectable antibody responses in mice and rabbit and sera from these immunized animals were used to characterize these antigens. All 21 proteins localized to the sporozoite: five localized to the surface, seven localized to the micronemes, cytoplasm, endoplasmic reticulum or nucleus, two localized to the surface and cytoplasm, and seven remain undetermined. PBMC from RAS-immunized volunteers elicited positive *ex vivo* or cultured ELISpot responses against peptides from 20 of the 21 antigens.

**Conclusions:**

These T cell and antibody responses support our approach of using reagents from RAS-immunized subjects to screen potential vaccine antigens, and have led to the identification of a panel of novel *P*. *falciparum* antigens. These results provide evidence to further evaluate these antigens as vaccine candidates.

**Trial Registration:**

ClinicalTrials.gov NCT00870987 ClinicalTrials.gov NCT00392015

## Introduction

*Plasmodium falciparum* malaria develops from the bite of infected mosquitoes that deposit sporozoites into the skin leading to invasion and development in hepatocytes. A vaccine is urgently needed, and intervention at the sporozoite or liver stages (pre-erythrocytic) of the parasite life cycle has the potential to prevent both clinical disease and transmission. Malaria vaccine development has mostly been based on a small number of antigens that are thought to represent immunity induced either by natural transmission or whole parasites. Because sporozoites cannot be cultured, and optimal culture of liver stages requires primary human hepatocytes, the discovery and characterization of new protective pre-erythrocytic stage antigens is a major challenge.

Immunization with radiation-attenuated sporozoites (RAS) delivered by the bite of infected mosquitoes induces sterile protection in mice [1, 2] and non-human primates [3], and up to 100% protection in humans [4, 5]. More recently, 100% protection has been achieved in humans by controlled human malaria infection (CHMI) using purified, irradiated *P*. *falciparum* sporozoites injected intravenously (PfSPZ Vaccine, Sanaria) [6], or whole sporozoites administered by mosquito bite under chloroquine treatment [7, 8]. In addition, genetically attenuated sporozoites have been shown to be potently protective in animal models [9] and immunogenic in humans but are waiting for efficacy testing in humans [10]. CD8+ T cells, particularly those containing interferon-gamma (IFN-γ) [11], recognizing peptides derived from pre-erythrocytic stage antigens such as the immunodominant CSP, are thought to be critical in RAS-induced protection in mice [1, 11–16] non-human primates [17] and humans [18]. To date, pre-erythrocytic stage malaria vaccine development has focused upon a very small fraction of the approximately 2000 proteins, which are thought to be expressed during these stages [19]. The most advanced candidate vaccine, RTS,S, is a protein subunit vaccine, based on CSP fused to hepatitis surface protein has been shown to elicit protection in Phase III clinical trials [20] that is thought to be mediated by anti-CSP antibodies and CD4+ T cells [21]. Other lead vaccine antigens include the cell-traversal protein for ookinetes and sporozoites (CelTOS) [22, 23], the thrombospondin-related adhesion protein (TRAP) [24, 25], Exp-1 [26] and its *P*. *yoelii* ortholog HEP17 [27], Pf16 [28], STARP [29] and LSA1 [30, 31].

These antigens or multiple novel antigens other than CSP may contribute to protection in humans immunized with the PfSPZ Vaccine [32–34]; specific cellular immune responses to CSP, TRAP, LSA1 were infrequent and lower than those recalled by stimulation with whole sporozoites, although correlation with protection was not examined as all subjects were protected [6, 35]. Additional studies indicated that RAS-induced protection is mediated by the sum of low level antigen-specific immune responses targeting potentially hundreds of pre-erythrocytic antigens [36–38]. For example, 100% protection can be induced by RAS in CSP-tolerized mice unable to mount any immune response to the CSP antigen [12]. These unidentified and untested, novel sporozoite and/or liver-stage antigens may be effective vaccine candidates when used either alone or in combination with known pre-erythrocytic stage antigens.

The publication of the *P*. *falciparum* genome [39] provided the sequence information required to conduct the antigenic analyses of genomic and proteomic data [38], enabling identification and development of novel malaria vaccines [40]. A variety of approaches for pre-erythrocytic antigen discovery have been undertaken [19, 38, 41–45]. In earlier studies, the characterization of proteins expressed in sporozoites by mass spectrometry [36] identified a panel of antigens as potential targets of the RAS-induced cellular responses in humans [38], and synthetic peptides from some of these proteins recalled IFN-γ activities from RAS-immunized volunteers that in some cases were higher than responses recalled by peptides of known antigens such as CSP [38].

We have previously reported the successful use of the Gateway system for the high throughput cloning of 254 *P*. *falciparum* single and multi-exon open reading frames [46] selected based on combination of bioinformatics analysis of various transcriptome and proteome expression databases for sporozoites [36, 47] and liver stages [42]. The gene selection process was previously described [46] in which a panel of genes were selected based on a comparative analysis of two sporozoite expression transcriptome [47] and proteome [36] databases. In addition, the selection of *P*. *falciparum* orthologs of *P*. *yoelii* liver stage genes was derived from a study identifying *P*. *yoelii* cDNA transcripts by laser micro-dissected liver stage parasites [42].

Attempts to express these clones presented additional challenges; the availability of an efficient protein expression platform is vital for vaccine development [48] and structural genomics studies [49, 50]. Although we were able to express only a small proportion of these proteins in *E*. *coli* (16%), some improvement was achieved when using a Gateway-compatible *E*. *coli* cell-free system, enabling expression of 17 of 39 (44%) proteins [46]. The low expression efficiency of *Plasmodium* recombinant protein in heterologous expression system, especially *E*. *coli* has been attributed to the amino acid composition of these proteins which has a bias towards amino acids coded predominantly by high AT genetic codons. Our analysis of expressed and non-expressed genes showed this bias was not the case [46]. The toxicity of nascent proteins to the bacteria is another culprit issue since most of the *Plasmodium* proteins are expressed non-soluble, truncated or not expressed at all. This argument is supported by our and others experience of a higher expressing efficiency when using a cell-free system, either *E*. *coli* [46] or wheat germ as shown in this study. The correct folding is another advantage of a eukaryotic system like the wheat germ over the prokaryotic cells, due to its low translation rate and co-translation protein-folding feature [51].

More recently, we used the modified wheat germ cell-free expression system [52, 53] to successfully express 131 of 151 (87%) proteins from a previously described gene set [46]. These recombinant proteins, expressed as both GST- and 6xHis-tagged fusions, were used to screen pools of plasma from RAS-immunized subjects (See Supplementary Information) as a first step to down-select a smaller panel of proteins for further characterization. Here we report the identification of 27 pre-erythrocytic *P*. *falciparum* proteins, of which 26 were recognized by RAS antibodies. Further cellular screening of these 27 proteins using overlapping peptides spanning predicted CD8+ T cell epitopes identified 20 antigens recalling ELISpot IFN-γ responses from these RAS-immunized subjects. We can only sample peripheral PBMC, and it is likely that liver-resident T cells contribute to RAS-induced protection [35]. CelTOS specifically recalled ELISpot IFN-γ activities from protected rather than non-protected volunteers, as suggested in a prior study using cells from the same RAS-immunization trial [37]. Mouse or rabbit antisera raised to the purified proteins demonstrated antigen expression and localization in *P*. *falciparum* sporozoites, liver, and blood stages. Our primary result is a list of qualified antigens to be considered for further development as malaria vaccines.

## Materials and Methods

### Ethics Statement

The study protocol for clinical specimens used in this research was conducted in compliance with all applicable Federal and Department of Defense regulations governing protection of human subjects. The clinical protocol was approved by the Naval Medical Research Center (NMRC) Institutional Review Board, the Office of the Special Assistant for Human Subject Protections at the Department of the Navy Bureau of Medicine and Surgery, and the Human Subjects Research Review Board of the Army Surgeon General. The study was conducted at the NMRC Clinical Trials Center in accordance with the principles described in the Nuremberg Code and the Belmont Report; all federal regulations regarding the protection of human subjects as described in 32 CFR 219 (The Common Rule) and instructions from the Department of Defense, the Department of the Navy, and the Bureau of Medicine and Surgery; and the internal policies for human subject protections and the standards for the responsible conduct of research of the US Army Medical Research and Materiel Command and NMRC. NMRC holds a Federalwide Assurance from the Office of Human Research Protections under the Department of Health and Human Services. NMRC also holds a Department of Defense/Department of the Navy Addendum to the Federalwide Assurance for human subject protections. All key personnel were certified as having completed mandatory human research ethics education curricula and training. All potential study subjects provided written, informed consent before screening and enrollment and had to pass an assessment of understanding.

All animal procedures reported herein were conducted under a protocol approved by the Institutional Animal Care and Use Committee at the Walter Reed Army Institute of Research/Naval Medical Research Center (protocol #D02-09) in compliance with the Animal Welfare Act and with the principles set forth in the “Guide for the Care and Use of Laboratory Animals,” Institute of Laboratory Animal Resources, National Research Council, National Academy Press 1996.

### Study Subjects

The PBMC and plasma samples used throughout this study were collected in 2000–2002 from 10 research subjects experimentally immunized with RAS delivered by mosquito bite over multiple immunization sessions to achieve >1000 infectious bites (Information of this trial is provided on the supporting information, S1 Text). Samples from three subjects mock immunized by similar numbers of bites of irradiated, non-infected mosquitoes were used as negative controls. The true-immunized subjects were challenged following five, six, or seven immunizing doses by bites of non-irradiated mosquitoes carrying infectious sporozoites; five of the 10 individuals were protected based on negative thin blood smears. Both PBMCs and plasma were collected from each individual before immunization (pre-immunization) and after the final (fifth, sixth, or seventh) immunizations, which were just before challenge (pre-challenge). Samples from this study have also been used in previously reported studies [37, 38].

### Study Subject HLA typing

HLA molecular typing for HLA-A and HLA-B loci was performed by the Department of Defense Bone Marrow Donor Program using specific oligonucleotide probes to amplify HLA Class I genes.

### Gene Selection and Cloning

All *P*. *falciparum* genes reported here were selected based on proteome and/or transcriptome datasets assigning gene expression in the sporozoite and/or liver stages of the parasite [46]. The 151 genes used in this study were previously selected and cloned in an earlier phase of this project [46]. For this study we have chosen the genes from a panel 159 genes based on their probability of expression in the pre-erythrocytic stages of malaria parasites. For sporozoite genes we used two expression databases; one based on transcriptome profiling [47] and the other on a proteomic analysis [36] of *P*. *falciparum* sporozoites and other stages. For liver stage parasites, we selected *P*. *falciparum* orthologs of genes identified from a cDNA library constructed with transcript laser micro dissected from *P*. *yoelii* liver stages parasites [42]. Eight clones out of the 159 genes could not be transferred to the protein expressed vector used in this study, and therefore, we screened 151 antigens. These 151 *P*. *falciparum* genes were cloned from this original panel using the Gateway cloning system (Invitrogen) into protein expression DNA constructs with GST- and 6xHis-fusions in the pEU-E01-GST-TEV-GW and pEU-E01-His-TEV-GW plasmids respectively [52]. These two plasmids had been converted to the Gateway system (labeled GW) and were kindly provided by Dr. Takafumi Tsuboi (Ehime University, Matsuyama, Japan). The batch sub-cloning was performed using the LR reaction protocol described earlier [46].

### Protein Expression

The expression of recombinant proteins by the wheat germ cell-free system was done at two scales each using a different protocol. All reagents and equipment were purchased from CellFree Sciences (Japan).

#### Small-Scale

Batch reactions were set up in 96-well U-bottom plates as described previously [54]. Briefly, the reaction was assembled by overlaying 40 μl of substrate mix (0.45 mg/ml creatine kinase, 20 units (U) of RNase inhibitor, 24 mM HEPES/KOH pH 7.8, 100 mM potassium acetate, 2.7 mM magnesium acetate, 0.4 mM spermidine, 2.5 mM dithiothreitol, 1.2 mM ATP, 0.25 mM GTP, 16 mM creatine-phosphate, 0.005% NaN_3_, and 0.3 mM of each of the amino acids including [14C]leucine (2 μCi/ml)) over 10 μl of translation mix containing 2 μl of each RNA clone and 8 μl of wheat germ extract OD240 (OD60 final concentration). Plates were covered with parafilm and incubated at 26°C for 16 hours.

#### Large-Scale

Large amounts of recombinant proteins were synthesized following general guidelines recommended by the wheat germ extract manufacturer. This expression was performed either manually or automatically using a workstation Protemist DTII (CellFree Sciences) designed for bilayer synthesis. The Protemist DTII is programmed to generate mRNA, express, and then affinity-purify recombinant proteins. Depending on the specific protein quantity required for various experiments, variable numbers of reactions were set up using flat bottom 6-well plates or the workstation. In the manual protocol, each translation reaction contained a bilayer of 2 mixtures; the lower portion included a total of 500 μl of the transcribed RNA and the wheat germ extract and the upper mixture consisted of 5.5 ml of dialysis buffer. The lower reaction mixture was prepared by mixing 250 μl of the RNA reaction, 40 mg/ml creatine kinase and 120 OD/ml of wheat germ extract (WGE OD240, CellFree Sciences). This lower mixture was transferred to a 6-well plate and carefully overlaid with 5.5 ml of sub-mix buffer supplied by the manufacturer in order to form a bilayer reaction. Plates were sealed with parafilm and translation was performed at room temperature overnight. Proteins were expressed in the Protemist DTII according to the manufacturer’s instructions.

### Protein Purification

Recombinant proteins tagged with GST were affinity-purified using a standard protocol [52]. After completion of the translation reaction, the product was absorbed into Glutathione Sepharose 4B resin (GE Healthcare) followed by protein elution with 20U of tobacco etch virus protease (AcTEV, Invitrogen) and 1 mM of DTT. Eluted proteins were confirmed by SDS-PAGE stained by Coomassie brilliant blue or silver stains. Purified protein concentrations were determined by a Bradford protein assay kit (Bio-Rad Laboratories). Proteins expressed as 6xHis fusion were affinity purified using Nickel-NTA agarose resin (Qiagen), eluted by imidazole in the Protemist DTII workstation.

### Animal Immunizations

#### Mice

Groups of two BALB/c or CD1 mice were immunized by subcutaneous (SC) injection three times at 3-week intervals with 20 μg of purified recombinant protein emulsified in Montanide ISA720 adjuvant (SEPPIC), at a 1:3 volumetric ratio of protein to adjuvant. Mice immunized with adjuvant alone (a 1:3 volumetric ratio of protein diluent to adjuvant) served as negative controls. Sera samples were collected two weeks after each immunization and were tested for activity to sporozoites by IFA.

#### Rabbits

Two albino rabbits weighing between 2 and 2.5 kg each (housed at Spring Valley Laboratories) were immunized per protein. Animals were immunized by SC injection three times at 3-week intervals with 50 μg of affinity-purified recombinant protein emulsified in Freund’s adjuvant (Sigma-Aldrich).

### Immunogenicity Assays

#### Immunofluorescence Antibody Assay (IFA)

Sera from both mice and rabbits immunized with expressed proteins, as well as monoclonal antibody (MAb) 2A10 to CSP [55], were tested by IFA for reactivity to sporozoites, 7-day *in vivo* liver stages, and asexual erythrocytic stages of *P*. *falciparum*, as previously described [56]. In brief both salivary gland sporozoites and *in vitro* cultured 3D7 *P*. *falciparum* parasites were used to prepare IFA slides, air dried and frozen at -80C till assay was performed. Liver stage sections were generated from frozen 7-day *P*. *falciparum*-infected chimeric livers [56]. Serial dilutions of the sera were incubated for one hour at 37°C, washed and developed with FITC-labeled goat anti-mouse or rabbit for 30 minutes at 37°C in the presence of Evan’s blue as a counterstain and to suppress any auto-fluorescence in the tissue. Specific reactivity were determined under a fluorescence microscope and recorded as digital pictures.

#### Immuno-Electron Microscopy

The localization of antigens in *P*. *falciparum* sporozoites was examined by immuno-electron microscopy using antigen-specific mouse or rabbit polyclonal sera or MAb 2A10. *P*. *falciparum*-infected salivary glands were dissected from *Anopheles stephensi* mosquitoes 14 days after a parasite-infected blood meal, and fixed in PBS containing 1% paraformaldehyde and 0.1% glutaraldehyde, for 24 hours at 4°C. Glands were embedded in LR-White resin (Polyscience), then ultrathin sections were cut and placed on nickel grids. The sections on the grids were etched by incubation with freshly prepared, saturated sodium-m-periodate for 5 minutes, followed by rinsing 3 times in deionized water. The grids were quenched with 0.1 M glycine in phosphate buffer for 20 minutes to prevent any free aldehyde groups from binding to the primary antibody. The grids were blocked by incubation in PBS, 1% BSA, 5% fish gelatin (Ted Pella) for 30 minutes. Grids were incubated with the primary test sera (diluted 1:50) in a humidified environment for 2 hours, followed by washing 5 times in PBS-0.1% Tween-20. The grids were then incubated for 30 minutes with a goat anti-mouse or goat anti-rabbit antibody conjugated to 10 nm gold particles (Ted Pella). The grids were washed as described above, then post-stained with 2% uranyl acetate and rinsed with water. The sections were examined with a 100 EX transmission electron microscope (JEOL USA). Negative controls included uninfected salivary glands and the use of nonspecific, irrelevant antibodies as the primary antibody.

#### Radio and Western Blots

^14^C-Leucine-labeled proteins were separated by SDS-PAGE as three fractions; 2 μl of the total translation reaction (T), and after the 96-well plate was spun, 2 μl of supernatant (S) fraction and 2 μl of the resuspended pellet fraction (P) were mixed in sample buffer. Recombinant proteins were identified by autoradiography using an imaging analyzer (BAS-2500; Fujifilm). Affinity-purified recombinant proteins were also screened by Western blot: 5 μg of each protein were separated on a 4–20% gradient SDS polyacrylamide gel (Invitrogen), and were subsequently electro-transferred onto a PVDF membrane (Millipore) and probed with a 1:500 dilution of rabbit or mouse antisera generated by protein immunization or with a 1/500 dilution of human RAS-immune sera. Peroxidase-conjugated goat anti-rabbit, anti-mouse, or anti-human IgG antibody (KPL) was used as the secondary antibody at a 1:10,000 dilution. The reaction was developed using an ECL-Plus Western blotting detection system (KPL) according to the manufacturer’s instructions.

#### *Ex vivo* ELISpot interferon-gamma (IFN-γ)

Previously frozen PBMC from subjects immunized with RAS were collected after the seventh RAS-immunization (two weeks post-immunization: subjects v20, v43, v58, v64 and v65; three weeks post-immunization: subject v30; four weeks post-immunization: subjects v52 and v53). Cells were stimulated with 15mer peptides overlapping by 11 amino acids, spanning each antigen (full-length), that were resuspended in DMSO as one pool with equal amounts of each peptide and tested at 10 μg/ml (final concentration of each peptide), or as separate pools of 10 peptides representing the HLA A and B types for each RAS-immunized volunteer (S1 Table). Positive responses to CSP and CelTOS were defined using three criteria as described previously [57]: (1) a statistically significant difference (p = <0.05) between the average number of spot forming cells/million PBMC (sfc/m) in triplicate test (pre-challenge) wells and the average of negative control (pre-immunization) wells (Student’s two tailed t-test), plus (2) at least a doubling of sfc’s in test (pre-challenge) wells relative to negative control (pre-immunization) wells, plus (3) a difference of at least 10 spots between test (pre-challenge) and negative control (pre-immunization) wells. However, because responses to novel antigens were low, we used a second different lower stringency definition of positivity used with PBMC from subjects exposed to natural malaria transmission in Ghana, where activities were also low [58]: a difference of at least 20 sfc/m between test (pre-immunization) and control (pre-challenge) wells. Some pre-immunization samples had high recall activities for reasons that are not clear but have also been observed in other vaccine trials [57].

#### Cultured IFN-γ ELISpot

The cultured ELISpot was performed as previously described [59] and reported by others [60]. Cryopreserved PBMCs from the RAS-immunized volunteers (2x10^6^ cells/ml) were plated in 100 μl triplicates in 96-well U-bottom plates (Corning) and stimulated with peptide pools of 1.25 μg/peptide/ml final concentration for each antigen. Recombinant human interleukin-2 (IL-2) at 10 U/ml and interleukin-7 (IL-7) (Life Technologies) at 10 ng/ml (10–50 U/ml) were added to the cell/peptide suspension and incubated in a 37°C, 5% CO_2_ humidified atmosphere. On day 12, cells were washed three times and resuspended in two ml of HR10 media. Cultured cell suspension containing 2x10^6^ cells/ml were plated for ELISpot assays as described for *ex vivo* ELISpot above (including stimulated cells and unstimulated controls for each cultured peptide stimulation), and assayed after 18 hours incubation. Positive activities were defined using the criteria as described for *ex vivo* ELISpot above [57].

## Results

### Protein expression

**Fig 1:**
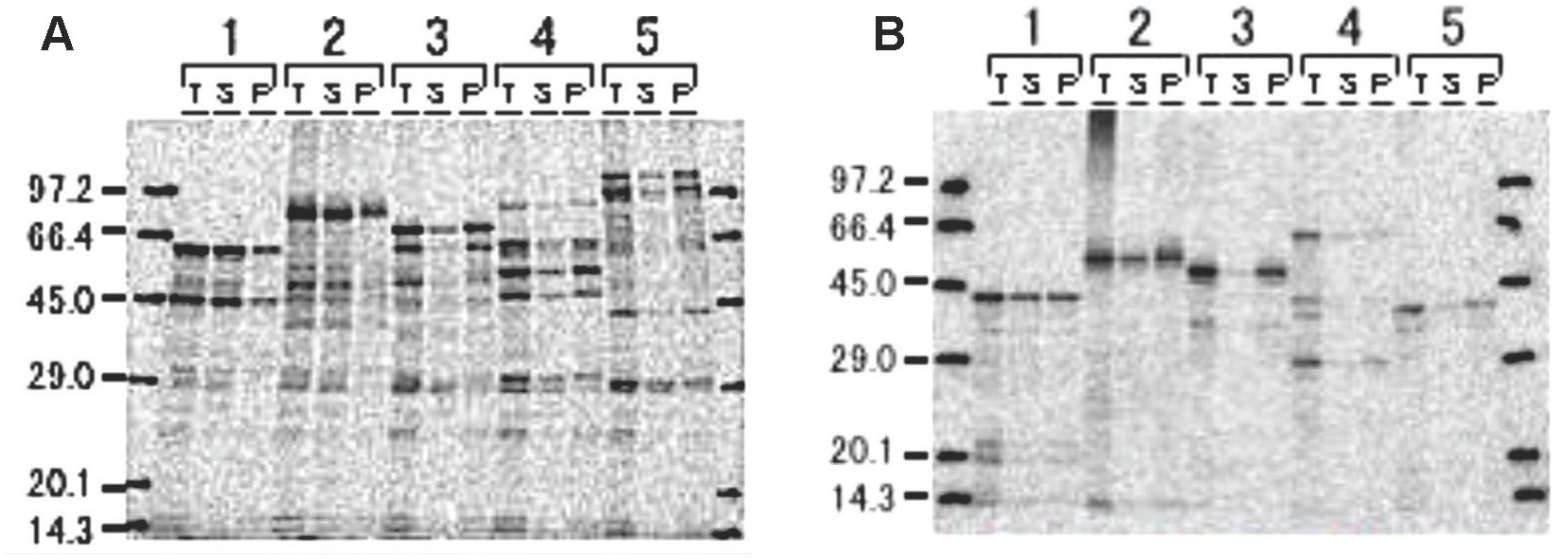
High-throughput expression and detection of *P*. *falciparum* proteins. Compartmental wheat germ expression of recombinant proteins represented by five clones as GST (A) and 6xHis fusions (B). [C14]-Leucine labeled proteins detected by radio blot in three fractions; total (T), supernatant (S), and pellet (P). Protein predicted molecular weight sizes are: (1) 32.8 kDa, (2) 50.2 kDa, (3) 37.7 kDa, (4) 59.7 kDa and (5) 32.5 kDa. The molecular sizes for GST-fused proteins (A) include an additional 29 kDa (GST molecular size). The autoradiographs for all the 151 clones are shown in S1 Fig.

*P*. *falciparum* proteins identified for this analysis were originally identified by high-throughput screening using the Gateway cloning system [46]. Initially, a small-scale protocol using a wheat germ cell-free system was used to express all 151 proteins of this original panel [46] (see Methods) as both GST and 6xHis fusion proteins. Overall efficiency was high, with 75% of GST and 72% of 6xHis fusion proteins expressed at their predicted molecular weight as determined by autoradiographs. In general, expression efficiency was not related to the size of the protein over a large range (8–127 kDa), although fewer 80–100 kDa proteins (8/23) were successfully expressed. The overall expression efficiency of these proteins was even higher (131/151, 87%) when truncated recombinant proteins were included. Expression and autoradiographs of the first five representative GST and 6xHis fusion proteins are shown in Fig 1, and the complete range of 151 proteins is shown in S1 Fig.

### Antigenicity of expressed novel *P*. *falciparum* proteins

#### Antibody screening

**Fig 2:**
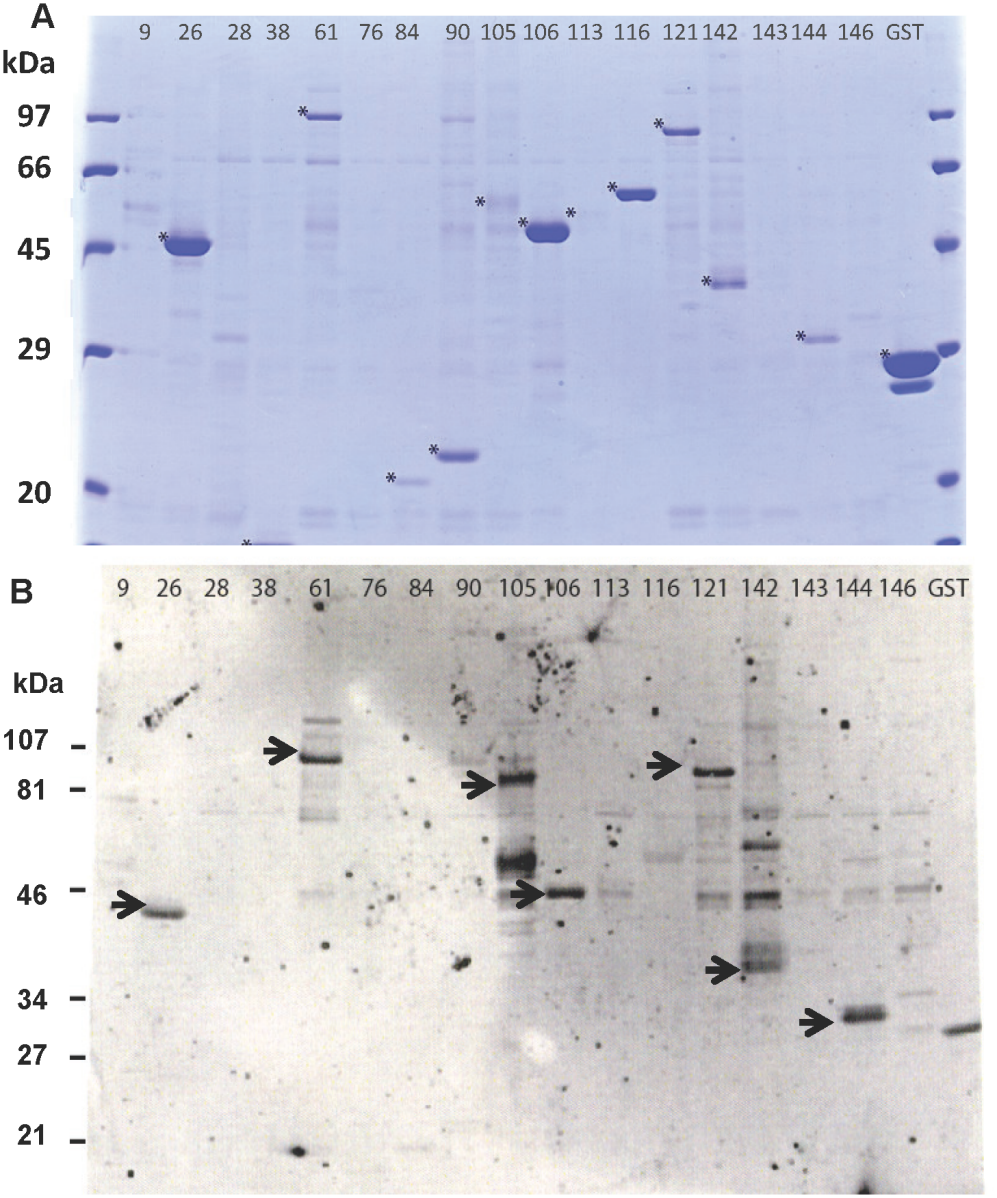
Expression and detection of affinity purified *P*. *falciparum* proteins. **(A)** Coomassie blue stained SDS-PAGE gels of 17 randomly-selected GST-fusion proteins that were affinity purified and cleaved. Asterisk indicates purified protein. (**B**) Western blot probed with pooled RAS-immune sera. Arrow indicates positive reactivity.

An initial antibody screening was performed to identify proteins definitively expressed during RAS-immunization, in order to increase the efficiency of the planned comprehensive characterization. Of the 151 recombinant proteins, 131 were expressed and affinity purified at large-scale and probed by Western blot for reactivity to a pool of plasma from RAS-immunized subjects. Of these 151 proteins, only 27 proteins were positive in Western blots when probed with the RAS plasma pool, and these were then selected for further characterization and cellular screening. Twenty-six of twenty-seven proteins were initially recognized; a representative Western blot is shown in Fig 2 depicting 17 randomly selected clones. One protein (Pf78), initially recognized by the pooled RAS plasma, was included in the down-selected list for characterization. However, in a subsequent screening by Western Blot, Pf78 was not positive, and therefore 26 proteins were recognized.

#### Characterization of 27 novel *P*. *falciparum* proteins

We next raised polyclonal sera to the 27 down-selected antigens for cellular and subcellular localization studies using the immunofluorescence antibody assay (IFA) and immuno-electron microscopy. Sera were raised in rabbits or mice by immunizing with affinity-purified recombinant protein emulsified in Freund’s and Montanide adjuvants, respectively. As positive controls, rabbit sera raised to CelTOS and anti-CSP MAb 2A10 [55], were used. IFAs were performed on sporozoites, infected chimeric human hepatocytes, and infected human red blood cells, while immuno-electron microscopy was only performed on sporozoites.

#### Known *P*. *falciparum* antigens CSP and CelTOS

**Fig 3:**
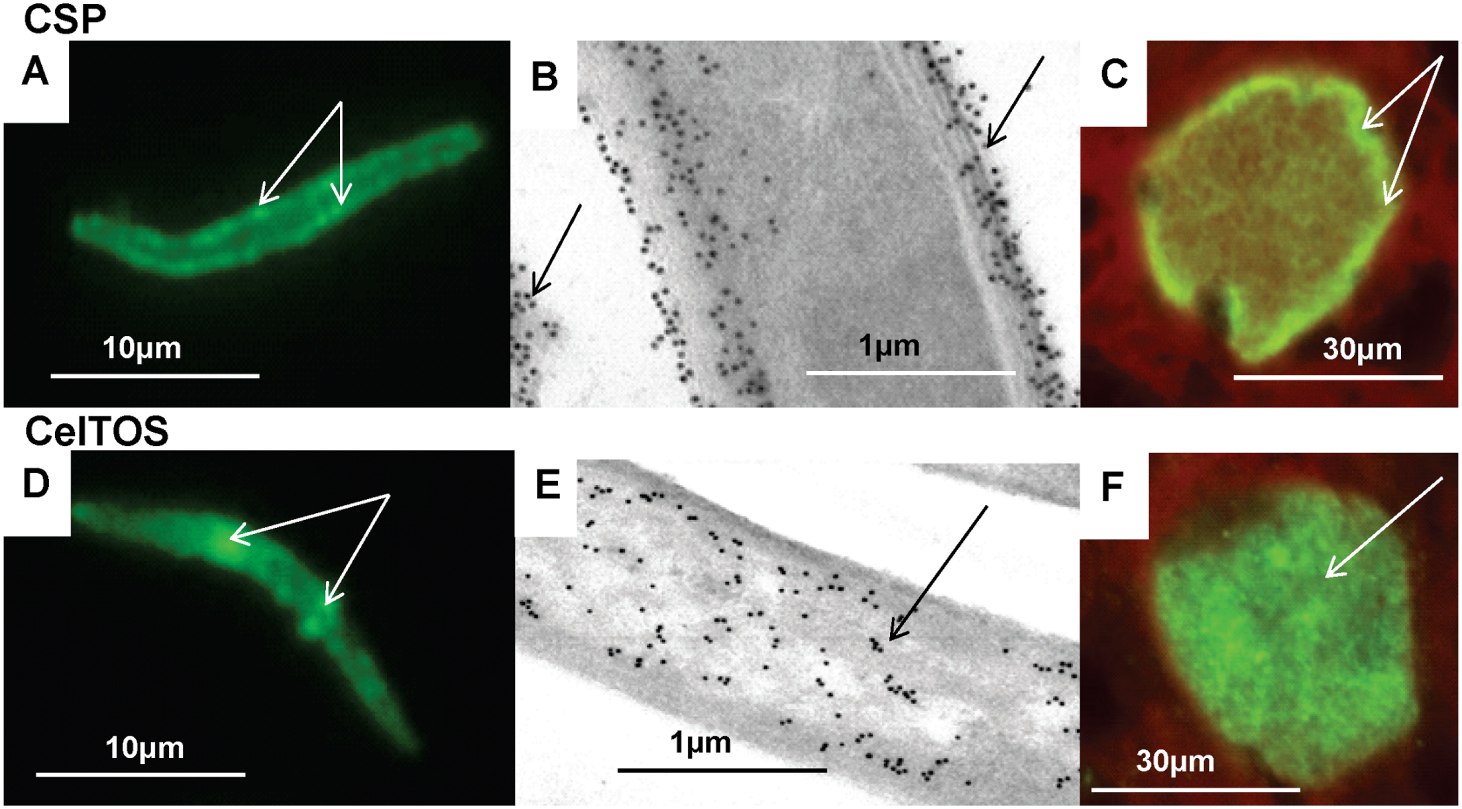
Stage-specific expression of CSP and CelTOS by immunofluorescence IFA and immuno-electron microscopy. CSP and CelTOS were localized to sporozoites (A, B, D, and E) and 7-day old liver stages (C and F) by IFA (A, C, D, F) and by immune-electron microscopy (B, E). **CSP**: localized to the sporozoite surface, (arrows, A), outer sporozoites membranes and shed material (arrows B), and the periphery of 5 day liver stage (arrows, C). **CelTOS**: localized to patches within sporozoites (arrows, D) that are associated with micronemes (arrows, E), was not on the surface, and was in the cytoplasm of five day liver stages (arrow, F).

We first characterized the reactivity of CSP and CelTOS as positive controls in our assays and to compare localization of our novel antigens with these well-known antigens. By IFA, CSP appeared to localize to the sporozoite periphery, whereas CelTOS appeared to localize to the sporozoite interior (Fig 3) in agreement with previous studies [2, 22]. This distribution was confirmed using immuno-electron microscopy (Fig 3): CSP evenly localized to the outer plasma membrane, inner pellicular membranes and internal micronemes, as previously described [61], and to material shed from the sporozoite, also consistent with a previous report [62]. CelTOS localized predominantly to micronemes as previously reported [22, 63]. CSP was detected in liver stages, but was not detected beyond 5 days post-infection as previously reported [64], and was predominantly localized to the parasite periphery. We also demonstrated for the first time that CelTOS was also present in liver stage development, and was detected five days following sporozoite infection (Fig 3).

#### Novel *P*. *falciparum* antigens

The results of IFA are shown as positive, negative, or not tested (Table 1). IFAs were performed using antisera raised to 21 antigens, as six failed to induce antibodies in animals (scored as ND, Table 1). All of the remaining 21 sera were positive with sporozoites. Sixteen sera were tested against *P*. *falciparum* 7-day liver stages, of which 15 were positive; none of these sera were only positive with liver stages. All 21 sera to these proteins were tested with mature blood stages, of which 13 were positive and none were positive to blood stages only. Negative controls were pre-immune sera that were consistently negative on these assays (data not shown).

**Table 1:** Summary of properties of 27 novel *P*. *falciparum* proteins.

Clone	RAS Sera	IFA SPZ	IFA LS	IFA BS	SPZ localization	*Ex vivo*	Cultured
	Blot					ELISpot	ELISpot
**Pre-erythrocytic (Sporozoites or Sporozoites/Liver Stages)**							
**Pf01**	**+**	**+**	**+**	**-**	NT	NT	**+**
**Pf24**	**+**	**+**	NT	**-**	Cytoplasm	NT	**+**
**Pf26**	**+**	**+**	**+**	**-**	NT	**+**	NT
**Pf47**	**+**	**+**	NT	**-**	NT	NT	NT
**Pf72**	**+**	**+**	NT	**-**	NT	NT	**+**
**Pf77**	**+**	**+**	**-**	**-**	NT	NT	**+**
**Pf83**	**+**	**+**	NT	**-**	NT	NT	**+**
**Pf116**	**+**	**+**	**+**	**-**	Surface	**+**	NT
**Sporozoites, Liver Stages, and Blood Stages**							
**Pf02**	**+**	**+**	**+**	**+**	Surface membranes	**-**	NT
**Pf08**	**+**	**+**	**+**	**+**	Surface	**-**	**+**
**Pf09**	**+**	**+**	**+**	**+**	NT	**+**	NT
**Pf13**	**+**	**+**	**+**	**+**	Surface, interior	NT	**+**
**Pf43**	**+**	**+**	NT	**+**	NT	NT	NT
**Pf56**	**+**	**+**	**+**	**+**	Surface clumps, cytoplasm	**+**	NT
**Pf61**	**+**	**+**	**+**	**+**	Cytoplasm	**+**	NT
**Pf78**	**-**	**+**	**+**	**+**	Inner pellicular membrane	**-**	**+**
**Pf84**	**+**	**+**	**+**	**+**	Surface clumps	**+**	NT
**Pf106**	**+**	**+**	**+**	**+**	Interior vesicles	**+**	NT
**Pf119**	**+**	**+**	**+**	**+**	Nucleus	**-**	**-**
**Pf121**	**+**	**+**	**+**	**+**	Interior vesicles	**+**	NT
**Pf144**	**+**	**+**	**+**	**+**	Micronemes, ER	**+**	NT
**Localization Not Determined**							
**Pf49**	**+**	ND	ND	ND	ND	NT	NT
**Pf51**	**+**	ND	ND	ND	ND	NT	**+**
**Pf59**	**+**	ND	ND	ND	ND	NT	NT
**Pf68**	**+**	ND	ND	ND	ND	NT	NT
**Pf93**	**+**	ND	ND	ND	ND	**-**	**+**
**Pf131**	**+**	ND	ND	ND	ND	NT	**+**

RAS = irradiated sporozoites; Blot = Western Blot; + = positive; - = negative; ER = Endoplasmic Reticulum; SPZ = Sporozoite; LS = Liver Stage; BS = Blood Stage; NT = Not Tested; ND = Not Determined (proteins were not sufficiently immunogenic to induce antibody responses).

Based on IFA results, the localization of these new antigens can be divided into three groups (Table 1): pre-erythrocytic (identified in sporozoites or in both sporozoite and liver stages): Pf01, Pf24, Pf26, Pf47, Pf72, Pf77, Pf83, and Pf116; all stages (identified in sporozoite, liver, and blood stages): Pf02, Pf08, Pf09, Pf13, Pf43, Pf56, Pf61, Pf78, Pf84, Pf106, Pf119, Pf121, and Pf144; since we were unable to generate antibodies in rabbits or mice to Pf49, Pf51, Pf59, Pf68, Pf93, and Pf131, the localization of these antigens remains undetermined.

**Fig 4:**
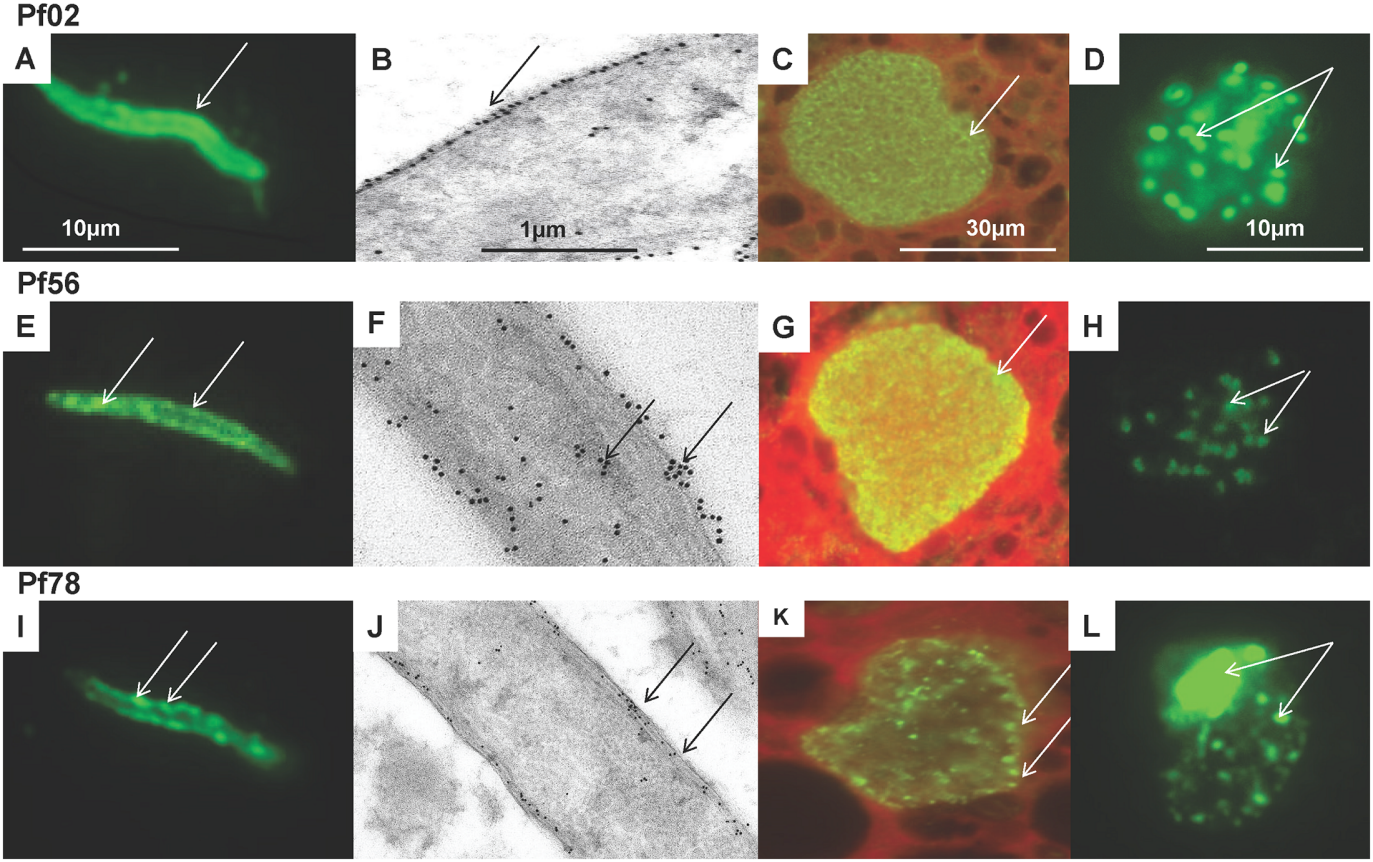
Stage-specific expression of three novel *P*. *falciparum* antigens by immunofluorescence and immune-electron microscopy. Three novel *P*. *falciparum* antigens (Pf02, Pf56, Pf78) were localized by immunofluorescence to sporozoites (A, E, I), 7-day liver stages (C, G, K), and blood stages (D, H, L); and by immuno-electron microscopy to sporozoites (B, F, J). **Pf02**: localized to the sporozoite surface (A), specifically the outer membrane (arrow, B), the cytoplasm of liver stages (C) and cytoplasm of blood stage merozoites (arrows, D). **Pf56**: localized to patches in sporozoite periphery (arrows, E, F) and sporozoite interior (arrows, F), the cytoplasm of liver stages (arrow, G) and cytoplasm of blood stage merozoites (arrows, H). **Pf78**: localized as discrete clumps on the sporozoite periphery (arrows, I), that were mostly in the middle and inner surface membranes (arrows, J), as discrete clumps in the liver stage periphery (arrows, K), and in the blood stage schizonts residual body and cytoplasm, but not merozoites (arrows, L).

**Fig 5:**
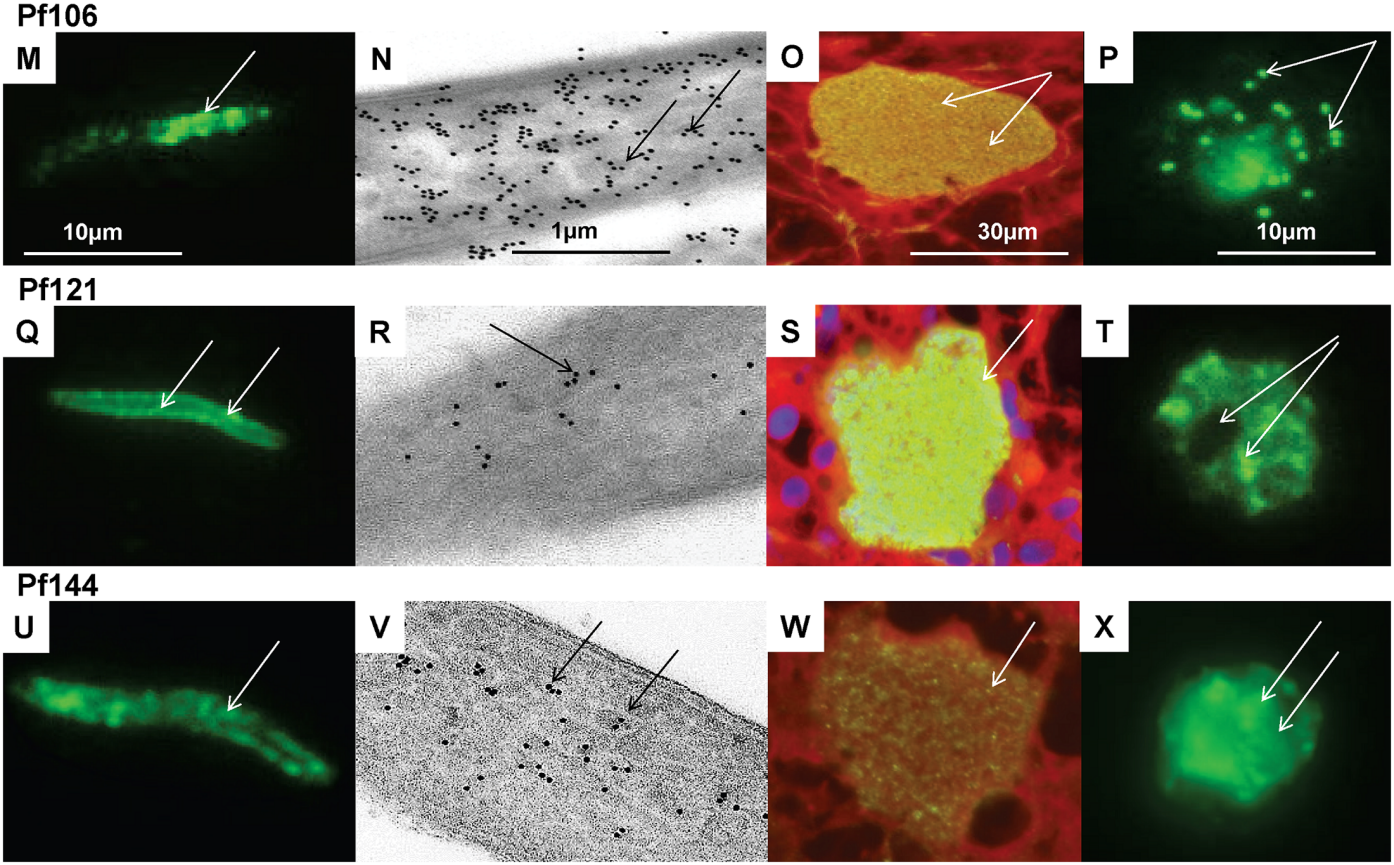
Stage-specific expression of three novel *P*. *falciparum* antigens by immunofluorescence and immune-electron microscopy. Three additional novel *P*. *falciparum* antigens (Pf106, Pf121, Pf144) were localized by immunofluorescence to sporozoites (M, Q, U), 7-day liver stages (O, S, W), and blood stages (P, T, X); and by immuno-electron microscopy to sporozoites (N, R, V). **Pf106**: localized in clumps at the sporozoite anterior pole (arrow, M), in vesicles inside the sporozoite (arrows, N), in liver stage merozoites (arrows, 0), and in blood stage merozoites (arrows, P). **Pf121**: localized unevenly in the sporozoite periphery/interior (arrows, Q) that appear to localize to micronemes (arrows, R), throughout liver stage parasite (arrow, S) and in clumps in blood stage schizonts but not the residual body (arrows, T). **Pf144**: localized to clumps inside the sporozoite (arrow, U) that are associated with micronemes (arrows, V), but is only weakly detected in 7-day liver stage parasites (arrow, W) and is dispersed in the blood stage schizonts (arrow, X).

Representative examples of each localization pattern are shown in Figs 4 and 5: Pf02 appeared to localize to the sporozoite periphery and in the cytoplasm of 7-day liver stage parasites, around merozoites, and was heavily expressed in blood stage merozoites; Pf56 appeared to localize to sporozoite peripheral clumps, and throughout 7-day liver stage parasites and was weakly expressed in blood stage merozoites; Pf78 appeared to localize to discrete peripheral patches on the sporozoite periphery, in apparent vacuoles surrounding and within 7-day liver stage parasites, and in patches and the residual body within the blood stage schizont; Pf106 appeared to localize to the apical pole end of the sporozoite, and appeared to localize to both liver stage and blood stage merozoites; Pf121 appeared to localize to discrete peripheral and internal patches of the sporozoite, was strongly detected in liver stage merozoites, and was distributed in patches in the blood stage schizonts but differed from Pf78 as it was not present in the residual body; Pf144 appeared to localize to clumps within sporozoites, and similar to Pf106, appeared to localize to liver stage schizonts, but, unlike Pf106, was found diffusely in blood stage schizonts.

Of the antisera to 21 antigens, that were positive by IFA to sporozoites, we were only able to positively localize 14 of the antigens using immune-electron microscopy, as antisera to the remaining seven antigens were too weak to use in this assay. These fourteen proteins were localized within surface and interior compartments of *P*. *falciparum* sporozoites and could be divided into three groups depending on their distribution. Six representative examples are shown in Figs 4 and 5, and the full list is shown in Table 1. Seven proteins were localized to the sporozoite surface: Pf02 localized to the surface membranes and shed protein but was not detected in micronemes, Pf56 localized to surface patches as well as the sporozoite cytoplasm, and Pf78 was sparsely distributed in the sporozoite inner pellicular membranes (Fig 4); Pf08, Pf13, Pf84, and Pf116 were also localized to the sporozoite surface (not shown). The localization of these seven surface antigens differed from CSP (Fig 3). Six proteins were localized in the interior of sporozoites: Pf106 was localized to micronemes within the apical pole of the sporozoite and in occasional clumps on the sporozoite surface; Pf121 was weakly localized within the cytoplasm of sporozoites; and Pf144 was weakly localized to sporozoite micronemes and possibly endoplasmic reticulum, and not to surface membranes (Fig 5). In addition, Pf24 and Pf61 were localized to the cytoplasm, and Pf119 was localized within the sporozoite nucleus (not shown).

### RAS-induced T cell responses targeting *P*. *falciparum* antigens

To test whether the cell-mediated responses induced by RAS-immunization recognized the antigens (and thus whether the antigens may potentially contribute to RAS-induced immunity), we conducted ELISpot assays using PBMCs obtained by leukapheresis from subjects after seven RAS immunizations and prior to undergoing CHMI. Five subjects were fully protected against CHMI and five were not protected. Frozen PBMCs were available from three protected and all five non-protected subjects for this study.

#### CelTOS and CSP

**Fig 6:**
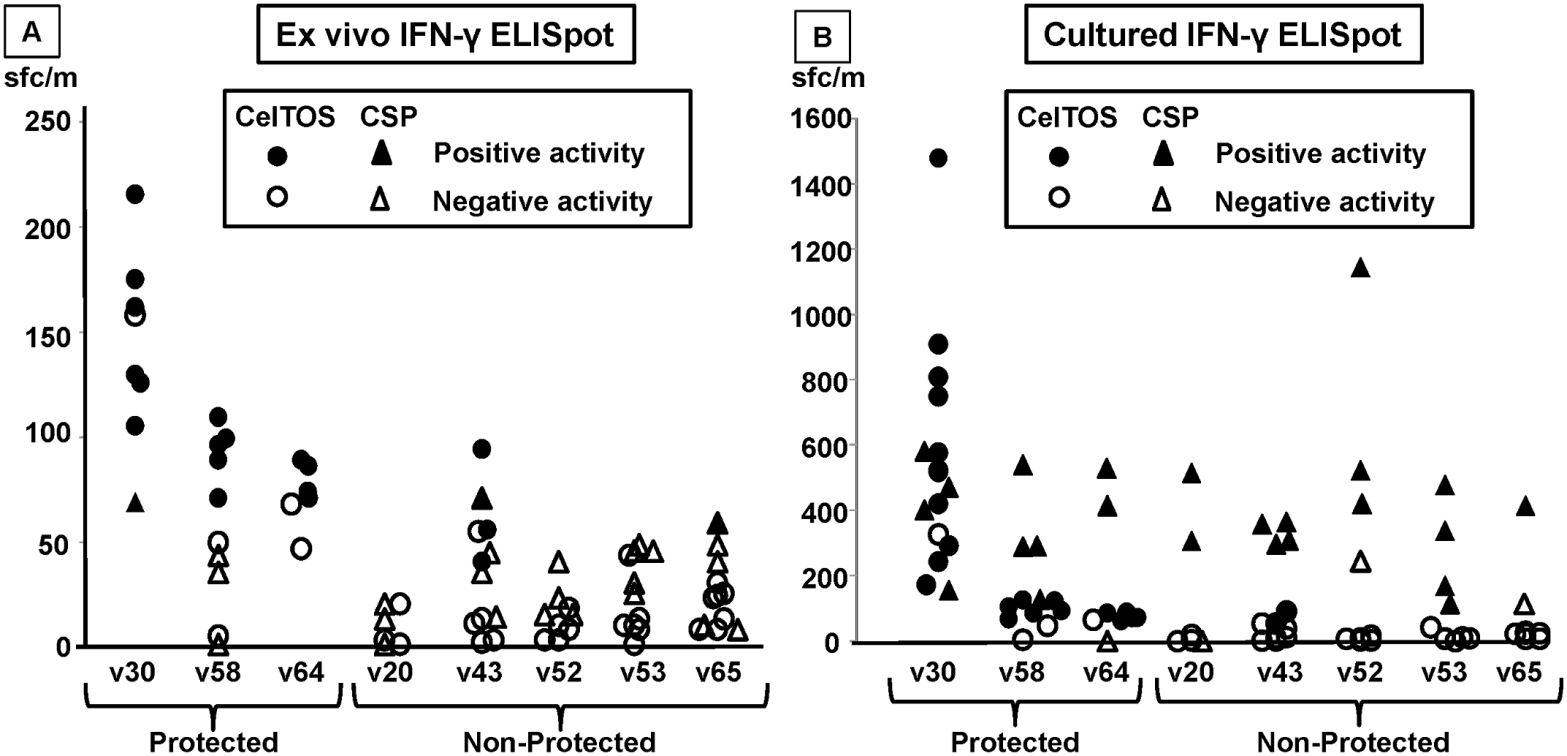
Antigen-specific IFN-γ ELISpot activity of protected and non-protected RAS-immunized volunteers targeting CelTOS and CSP. CelTOS (circles) and CSP (triangles) overlapping peptide pools spanning full length CSP or CelTOS were used to stimulate T cell responses of three protected (v30, v58, v64) and five non-protected (v20, v43, v52, v53, v65) RAS-immunized subjects, and responses were measured by IFN-γ ELISpot, and expressed as spot-forming cells/million PBMC (sf/m). **A**. In *ex vivo* assays, samples were scored as positive (filled markers) using high stringency criteria (significant difference between test antigen and medium controls using Student’s t-test, at least a doubling and a difference of at least 10 sfc/m (See Methods) or as negative (open markers). Occasional values of activities that were negative exceeded positive values if the pre-immunization activities were high. Overall, the CelTOS-specific T cell responses among protected subjects (geometric mean 98 sfc/m) were significantly higher than the CelTOS-specific T cell responses of non-protected subjects (geometric mean 12 sfc/m, *p* = <0.001, Mann-Whitney U test), whereas CSP-specific T cell responses were similar among protected (geometric mean 18 sfc/m) and non-protected (geometric mean 11 sfc/m, not significant) subjects. **B**. In cultured ELISpot assays, CelTOS-specific T cell responses of protected subjects (geometric mean 505 sfc/m) were also significantly higher than non-protected subjects (geometric mean 157 sfc/m, *p* = 0.002, Mann-Whitney U test). CSP-specific T cell responses of protected subjects (geometric mean 215 sfc/m) were similar to those of non-protected subjects (geometric mean 235 sfc/m, not significant).

We first tested peptides from *P*. *falciparum* CSP and CelTOS to determine that their recall activities were similar to those previously reported [38] despite being kept frozen for this extended period of time. Using *ex vivo* IFN-γ ELISpot, CelTOS recalled positive activities from all three protected subjects but one non-protected subject using the high stringency definition of positivity (see Methods) (Fig 6, Panel A). This definition of positivity was too stringent when novel antigens were used, where a lower stringency definition was used. Although PBMC were collected between two to four weeks post-immunizations (see Methods), there was no apparent correlation of these times and recall activities. The geometric mean of CelTOS-specific activity of protected subjects (98 sfc/m) was significantly higher (p = <0.001, Mann-Whitney U test) than non-protected volunteers (12 sfc/m). Fewer assays were conducted using CSP (Fig 6, Panel A), and recalled activities from one of two protected and two of four non-protected subjects. CelTOS previously to recalled similar activities (128±91 sfc/m) [38] to those reported here, confirming that these frozen PBMC could be used with our novel antigens.

To improve sensitivity of the assay, we also performed cultured ELISpot [65] using PBMCs from the same RAS-immunized subjects (Fig 6, Panel B). As observed with the *ex vivo* ELISpot, CelTOS recalled specific responses (using the same high stringency criteria as *ex* vivo ELISpot, Fig 6, Panel A) from all three protected subjects tested and five of five non-protected subjects tested (Fig 6, Panel B). The geometric mean activities of CelTOS-specific responses were higher in protected subjects (geometric mean 505 sfc/m) than in non-protected subjects (geometric mean 134 sfc/m, *p* = 0.002, Mann Whitney U test). CSP recall activities were detected in all three protected and five non-protected subjects (Fig 6, Panel B), but CSP-specific activities of protected subjects (geometric mean 215 sfc/m) were similar to those of non-protected subjects (geometric mean 235 sfc/m, not significant).

#### Novel *P*. *falciparum* antigens

Because *ex vivo* IFN-γ ELISpot activities in response to CelTOS and CSP peptide stimulation were low, we anticipated that the panel of novel antigens might also recall low frequency T cell responses. We have previously shown that a single pool of peptides representing full length CSP recalled lower activities than the same peptides tested as multiple separate pools [66, 67], some of which represented immunodominant epitopes [67]. Therefore, for each novel antigen, we tested both a single peptide pool containing all overlapping peptides for each antigen, as well as smaller pools of 15mer peptides containing MHC class I-restricted T cell epitopes predicted to match the HLA of the tested RAS-immunized subject, as was previously done in a separate malaria vaccine trial [67]. Because PBMC samples from RAS-immunized subjects were limited, we first tested 14 of the panel of 27 novel antigens in *ex vivo* ELISpot with PBMCs of three protected and four non-protected subjects (Table 2). Limited availability of PBMC also led us to first choose a representative sample of 14 antigens that were selected based on their confirmed presence in sporozoites and liver stages using immune-electron microscopy; five were localized on surface of sporozoites (Pf02, Pf08, Pf56, Pf84, Pf116), seven were localized inside sporozoites (Pf09, Pf61, Pf78, Pf106, Pf119, Pf121, Pf144), and two were not determined (Pf26, Pf93) (Table 1).

**Table 2:** Antigen-specific *ex vivo* ELISpot IFN-γ responses of RAS-immunized subjects targeting novel *P*. *falciparum* antigens.

		Protected Subjects			Non-Protected Subjects			
		v30	v58	v64	v20	v43	v52	v53
Pf clone	Peptides	A02/A03	A02/A03	A02/A03	A02/A03	A03/A26	A0103/A03	A01/A02
		B07/B44	B07/	B07/B27	B44/B62	B62/	B07/	B07/
**Pf02**	**A02**	18	5	13				33
**Pf08**	**A02**	1	13	0			0	
**Pf09**	**All**					10		5
	**A02**	0	**20**	0				18
	**B07**	18	3				**20**	
**Pf26**	**All**	15	**28**	13		3	20	5
	**B07**	0	18				8	**48**
**Pf56**	**All**	0	5	8	0	0	0	18
	**A01**					**33**		20
	**B62**				**27**	0		
**Pf61**	**All**	8	18	3		0	18	5
	**A02**	5	25	5	**23**			
	**A03**		**40**					
**Pf78**	**B07**	1	12	0			0	
**Pf84**	**All**					3	0	8
	**A02**	**23**	8	5		18		0
**Pf93**	**All**	4		1		8	3	0
**Pf106**	**All**		**38**	15	**38**	15		
	**A02**	15	15	18	**28**			
	**B07**	15	**35**				5	
**Pf116**	**All**	0	25	0		0	0	0
	**A02**	0	**25**	13	13			
**Pf119**	**All**	3		0		0		0
**Pf121**	**A03**	8	**25**					
**Pf144**	**A02**	15	8	15				
	**A03**	**30**	15	13		15		**28**
	**B07**	**45**	**35**				5	0

Peptide pools containing 15mer overlapping peptides spanning the full length (All) or predicted HLA-specific regions were tested in triplicate with pre-immunization and post RAS-immunization (pre-challenge) PBMCs. Numbers are the average of triplicate assays as sfc/m. Positive responses (in bold and larger font size) were defined as a difference of 20 sfc/m between pre- and post-immunization. Only peptide pools recalling positive responses are included. Antigen-specific cell responses exceeding the positive criteria in at least one volunteer were detected in response to stimulation by nine of 14 novel *P*. *falciparum* antigens using total or HLA-specific peptide pools. Since responses were low, cultured ELISpot IFN-γ responses were measured (Table 3).

None of the peptide pools recalled activities that were positive using the highly stringent positivity criteria as was used for CelTOS- and CSP-specific responses. However, we had previously used a lower stringency definition of positivity, namely a difference of 20 sfc/m between control and test wells, using PBMC from residents of malaria-endemic areas in Ghana, where activities were also low, and reproducibly identified positive subjects in replicate assays performed on different days (58). Using this less stringent definition, two of the 14 tested full-length antigen peptide pools (Pf26 and Pf106) recalled positive activities, but HLA-specific peptide pools of nine of 14 antigens were positive: Pf09 A02 and B07; Pf26 B07; P56 A01 and B62; Pf61 A02 and A03; Pf84 A02; Pf106 A02 and B07; Pf116 A02; Pf121 A03; and Pf144 A03 and B07; the remaining five antigens (Pf02, Pf08, Pf78, Pf93, and Pf119) were negative (Table 2). There was no apparent difference between recall activities in protected vs. non-protected subjects.

Since responses were low, we also used cultured ELISpot to better identify positive responses to test 12 novel antigens (Table 3) for recognition by the same protected and non-protected volunteers as tested previously by *ex vivo* ELISpot. Since PBMC were limited, we decided to expand the number of antigens that were tested in either ELISpot assay by including four antigens that were tested in ex vivo ELISpot (Pf08, Pf78, Pf93, Pf119) and eight additional antigens that were either localized inside sporozoites (Pf13, Pf24), or not localized (Pf01, Pf51, Pf72, Pf77, Pf83, Pf131) by immune-electron microscopy. We recognize that this may have not sufficiently tested all these antigens in both assays but we chose at the time to test as many as possible in either assay rather than focus on a more restricted number of antigens. PBMCs were stimulated with peptide pools and were cultured for 12 days prior to performing the IFN-γ ELISpot to assess the frequency of antigen-specific expanded T cell responses. As we observed for the CelTOS and CSP peptide pools (Fig 5, Panel B), antigen-specific T cell responses were high following expansion, and we therefore used the highly stringent criteria to define positive responses. Ten of the 12 tested full length peptide pools were positive at the post-immunization time point by comparison to the pre-immunization time point: Pf01; Pf08; Pf13, Pf24; Pf51; Pf72; Pf77; Pf78; Pf83; and Pf93, whereas Pf119 was negative, and Pf131 full length was not tested. Six of the 10 positive antigens were also positive using HLA-specific peptide pools: Pf01 B07; Pf13 A02 and B07; Pf51 B07 and B62; Pf72 A01A03, A02, B07 and B62; Pf77 A01; and Pf83 A02; and in addition Pf131 A02 was also positive (Table 3). Thus 11 of the 12 tested novel antigens were positive in one or more subjects.

**Table 3:** Antigen-specific cultured ELISpot IFN-γ responses of RAS-immunized subjects targeting novel *P*. *falciparum* antigens.

		Protected Subjects			Non-Protected Subjects				
		v30	v58	v64	v20	v43	v52	v53	v65
Pf clone	Peptides	A02/A03	A02/A03	A02/A03	A02/A03	A03/A26	A0103/A03	A01/A02	A01/A24
		B07/B44	B07/	B07/B27	B44/B62	B62/	B07/	B07/	B08
**Pf01**	**All**	**540**	**558**	370	**192**	203	347	303	**293**
	**B07**	**222**	0	93	75	67	1	3	
**Pf08**	**All**	205		**207**		145	44	7	217
**Pf13**	**All**	**557**	13	5	**88**	**240**	192	13	
	**A02**	0	**120**	**67**	23		**237**	16	
	**B07**	70	3	9	57	5	**392**	5	
**Pf24**	**All**	63	240	**392**	1	280	82	222	**345**
**Pf51**	**All**		172	**283**		**232**		170	**153**
	**B07**	**208**	**68**	7					
	**B62**				**225**	0			
**Pf72**	**All**	185	0		0	198	**567**	57	
	**A01A03**					33	**97**		
	**A02**	30	0	0	**88**				
	**B07**	**143**	1	**327**	23		2		
	**B62**					**72**			
**Pf77**	**All**	**280**	**92**	**130**	15	**177**		170	**153**
	**A01**					**97**			
**Pf78**	**All**	263	**468**	142	208	**1087**	1	29	**322**
**Pf83**	**All**	250	3	52	1	1	**75**	8	
	**A02**	40	5	**123**	3		**27**	1	
**Pf93**	**All**	185	200	148		240	**303**	28	34
**Pf119**	**All**	198	257	198	61	150	36	27	
**Pf131**	**A02**	**100**	62	48	57		**75**		

Peptide pools containing 15mer overlapping peptides spanning the full length (All) or predicted HLA-specific regions were tested in triplicate with pre- and post-RAS immunization (pre-challenge) PBMCs in triplicate. Numbers are the average of spot forming cells/million PBMCs (sfc/m). Positive responses (in bold and large font size) were defined as a statistically significant difference (*p* = <0.05) between the average of the number of sfc/m in triplicate test (post-immunization) wells and the average of negative control (pre-immunization) wells (Student’s two tailed t-test), plus (2) at least a doubling of SFCs in test wells relative to negative control wells, plus (3) a difference of at least ten spots between test (post-immunization) and negative control (pre-immunization) wells. Antigen-specific T cell responses exceeding the positive criteria in at least one volunteer were detected in response to stimulation by 11 of 12 Pf clones, using total or HLA-specific peptide pools. Only Pf119 was negative.

In total, 22 novel *P*. *falciparum* proteins were tested in *ex vivo* and/or cultured ELISpot assays and the results are shown in Table 1. Twenty of 22 novel antigens were positive in either *ex vivo* or cultured ELISpot. We interpret this as validating our experimental approach to identify novel *P*. *falciparum* antigens recognized by RAS-immunized subjects and therefore potentially contributing to protective immunity, but we are not suggesting that recall activities of these novel antigens are correlated with protection. Overall, we have identified 27 novel *P*. *falciparum* antigens recognized by plasma and/or T cells of RAS-immunized subjects, therefore potentially contributing to RAS-induced protective immunity. The putative properties of these 27 novel antigens are shown in Table 4. These novel antigens are attractive malaria vaccine candidates for further characterization. We observed no obvious relationship between antigen localization and antigen-specific ELISpot activities.

**Table 4:** Panel of 27 novel *Plasmodium falciparum* proteins.

	Clone	Gene Locus	Protein Description
1	Pf01	PF10_0098	Conserved *Plasmodium* Protein, unknown function
2	Pf02	PFE0785c	Glideosome-associated protein 40, putative (GAP40)
3	Pf08	PFC0555c	Conserved *Plasmodium* Protein, unknown function
4	Pf09	MAL7P1.32	DNA Repair Protein
5	Pf13	PFC0700c	Microneme associated antigen (MA)
6	Pf24	PFC1055w	Conserved *Plasmodium* Protein, unknown function
7	Pf26	PFI1425w	Transcription initiation factor TFIID subunit 7, putative (TAF7)
8	Pf43	PF14_0620	tRNA 3'-trailer sequence RNase, putative
9	Pf47	PF11_0156	Serine/threonine protein kinase, putative (CLK3)
10	Pf49	PFD0445c	Conserved *Plasmodium* Protein, unknown function
11	Pf51	PFE0565w	Conserved *Plasmodium* Protein, unknown function
12	Pf56	PF08_0008	GPI-anchored micronemal antigen (GAMA)
13	Pf59	PF14_0495	Rhoptry Neck Protein 2 (RON2)
14	Pf61	PF10_0138	Conserved *Plasmodium* Protein, unknown function
15	Pf68	PF14_0722	Cysteine repeat modular protein 4 (CRMP4)
16	Pf72	MAL13P1.25	Conserved *Plasmodium* Protein, unknown function
17	Pf77	MAL13P1.212	Sporozoite protein essential for cell transversal (SPECT)
18	Pf78	MAL8P1.78	Small Heat Shock Protein (HSP20)
19	Pf83	PFA0200w	Thrombospondin-related sporozoite protein (TRSP)
20	Pf84	PFD0430c	Sporozoite micronemal protein essential for cell traversal, Perforin-like protein 1 (PLP1)
21	Pf93	PF13_0012	Early transcribed membrane protein 13 (ETRAMP13)
22	Pf106	PFI0580c	Falstatin, Cysteine Protease Inhibitor (ICP)
23	Pf116	PFI0460w	Subpellicular microtubule protein 1, putative (SPM1)
24	Pf119	PFD0235c	Conserved *Plasmodium* Protein, unknown function
25	Pf121	PF10_0319	Conserved *Plasmodium* Protein, unknown function
26	Pf131	MAL13P1.107	Conserved *Plasmodium* Protein, unknown function
27	Pf144	PF14_0467	Gamete egress and sporozoite traversal protein, putative (GEST)

This panel is derived from the previously published clones (see reference number [47]) and the Pf numbers represent those proteins characterized in Fig 1.

## Discussion

The objective of this study was to identify and characterize novel *P*. *falciparum* antigens that contribute to protective immunity elicited by RAS in humans, and to prioritize their development as candidates for inclusion in pre-erythrocytic stage vaccines. While CD8+ T cells targeting CSP protect mice [1, 12] and non-human primates [17], non-CSP antigens have also been shown to contribute to protection [32, 33], but to date it has been difficult to systematically identify these antigens. We have successfully applied a novel protein expression platform, the wheat germ cell-free system [52, 53], to screen 151 *P*. *falciparum* proteins, and identified 26 involved in the antibody responses to RAS immunization. These were distinct from another panel of novel *P*. *falciparum* antigens that were down-selected by our program using bioinformatics analysis, multidimensional protein identification technology (MudPIT [68]), and HLA-binding predictions [38]. Common to both approaches was the screening of novel antigens using peptides representing each antigen to recall antigen-specific T cell responses producing IFN-γ from frozen PBMCs of the same RAS-immunized subjects [37, 38]. For comparison and validation of our approach, we also included two well-characterized sporozoite antigens, CSP [2] and CelTOS, [38] the latter identified as antigen 2 in an earlier study [38]. This allowed comparison of T cell responses across both studies, which were conducted 10 years apart.

The study reported here confirmed the value of the wheat germ expression system [53] for expressing novel *Plasmodium* proteins, as 136/151 (87%) clones (although fewer large proteins of 80–100 kDa) were successfully expressed. This allowed us to screen these proteins for reactivity to RAS-immune human sera and to raise antigen-specific mouse or rabbit antibodies to characterize our panel of 27 novel proteins. Of these, 26 (17%) were recognized by plasma from RAS-immunized subjects after repeated testing. By using antigen-specific immune sera to these antigens, we identified 21 novel antigens to be expressed in pre-erythrocytic stages, of which eight are expressed in sporozoites and liver stages only, and 13 are expressed by both pre-erythrocytic and blood stage parasites. Another important outcome of the immuno-electron microscopy experiments was the identification of seven antigens localized on the surface of sporozoites that could be targets of parasite invasion-blocking antibodies. These antigens were distinct from the CSP and CelTOS that in our immuno-electron microscopy analysis localized to the surface [2] and micronemes [22] of sporozoites, respectively. Of note, this analysis showed for the first time that CelTOS was expressed during liver stage development.

To confirm that these novel antigens potentially contribute to protective immune responses elicited by RAS, we tested their ability to recall cell mediated responses in a group of RAS-immunized subjects who were challenged by CHMI, where 5/10 (50%) were protected and 5/10 (50%) were not protected [37, 38]. Liver-resident PBMC are the likely predominant mediators of RAS-induced immunity [35, 69], and we recognize that recall activities of peripheral PBMC may under-estimate responses in the liver [35]. Of interest was the finding that, using the *ex vivo* ELISpot assay, peptides spanning full length CelTOS recalled significantly higher positive responses from protected than non-protected subjects. In contrast, CSP recalled similar low activities from both protected and non-protected subjects, which were similar to the low activities previously reported [38]. This confirms and extends the previous observation that T cell IFN-γ activities to CelTOS were associated with protection in subjects immunized by RAS delivered by mosquito bite [38], and indeed CelTOS induced up to 60% efficacy in BALB/c and CD1 mice [23] suggesting its potential as a malaria vaccine candidate. In contrast, in another study in which sporozoites were administered intravenously, CelTOS and five other pre-erythrocytic antigens including CSP recalled low or undetectable responses in ELISpot assays despite the fact that 100% of subjects receiving the highest dose of sporozoites were protected against CHMI [6]. In that study, recall responses were also demonstrated by stimulation with whole *P*. *falciparum* sporozoites, indicating that sporozoite-specific cell mediated responses were in fact induced, even while responses to CSP, CelTOS, and other previously characterized pre-erythrocytic antigens including TRAP and LSA1 were very low or undetectable [6]. Taken together, these data suggest that the mechanisms of protective immunity induced by RAS delivered by mosquito bite or by purified sporozoites administered intravenously may differ as CelTOS recalled activities from subjects immunized by mosquito bite but not by intravenous administration, and that non-CSP antigens may contribute differently to RAS-induced protective immunity in each type of RAS immunization.

Since recall responses using *ex vivo* ELISpot were low, we applied a lower stringency definition of positivity previously used in a study of natural transmission in Ghana where responses were also low [58] and we were able to identify nine novel antigens that recalled positive antigen-specific activities, but only using peptide pools of a subset of 15mers containing predicted HLA-restricted epitopes matching the tested volunteer. However, using cultured ELISpot assays, positive recall responses targeting CelTOS, CSP, and notably, to 11 novel *P*. *falciparum* antigens, including three antigens negative in *ex vivo* ELISpot, were much higher, and were induced by both peptide pools spanning the complete novel antigen and by HLA-specific peptide pools. Different T cell populations are likely measured by *ex vivo* and cultured ELISpot assays, and correlates of protective immunity may depend on the pathogen being studied [70]. Our studies suggest that nine of 14 novel *P*. *falciparum* antigens described here recalled weak *ex vivo* responses, whereas 11 of 12 novel *P*. *falciparum* antigens recalled cultured ELISpot responses and these novel antigens may contribute to inducing memory T cell IFN-γ responses in these RAS-immunized subjects. This may be important as RAS induce protective immunity that persisted for up to nine months [18].

The next step will be to determine whether these novel *P*. *falciparum* antigens can individually or in combination induce protection from malaria. As a first step, we have shown that a combination of two *P*. *yoelii* ortholog antigens of Pf93 and Pf106, when administered as gene-based vaccines, yielded 43% sterile protection in outbred CD1 mice compared to 14% using *P*. *yoelii* CSP [71]. Pf106 recalled IFN- γ-producing cell responses from RAS-immunized volunteers by *ex vivo* ELISpot and Pf93 recalled IFN-γ -producing cell responses by cultured ELISpot. The *P*. *yoelii* ortholog of Pf106, PY03424, has also been shown to recall low frequency responses by IFN-γ ELISpot in mice immunized with *P*. *yoelii* RAS [33].

Pf106 has been previously described as malaria protein Falstatin, a cysteine protease inhibitor that is secreted by sporozoites and is crucial for sporozoite motility during hepatocyte invasion [72, 73]. Pf106 is localized within sporozoite vesicles (Fig 4B) consistent with the demonstration that Falstatin co-localizes in secretory vesicles with another sporozoite protein, thrombospondin-related adhesion protein (TRAP) [72]. Falstatin is also essential for liver stage development [72] and may also be a target of protective CD8+ T cells elicited during immunization with the *P*. *yoelii* ortholog.

## Conclusions

Twenty-one novel sporozoite and liver-stage antigens have been identified that were recognized by plasma of RAS-immunized subjects and pre-erythrocytic expression was confirmed by localization studies using polyclonal sera specific for the recombinant proteins. Importantly, seven antigens were localized to the surface of sporozoites indicating that they may be a target to infection-blocking antibodies. Twenty of these pre-erythrocytic antigens recalled antigen-specific activities from RAS-immunized subjects, suggesting a potential role in RAS-induced T cell immunity. The *P*. *falciparum* antigen CelTOS recalled antigen-specific cell responses that were differentially detected among protected and non-protected subjects. These data, combined with the induction of protective immunity in mice using *P*. *yoelii* orthologs [71], provide compelling evidence to further evaluate and advance these novel antigens as antibody- or cellular-based vaccine candidates.

### Disclaimers

The authors thank all the study volunteers who participated in the trial. The views expressed in this article are those of the authors and do not necessarily reflect the official policy or position of the Department of the Navy, Department of Defense, nor the U.S. Government. This work was supported by funded by work unit number 62787A 870 F 1432. The study protocol was approved by the Naval Medical Research Center Institutional Review Board in compliance with all applicable Federal and DoD regulations governing the protection of human subjects. SLH, DC, EDV and TLR were military service members and MS was an employee of the U.S. Government. This work was prepared as part of my official duties. Title 17 U.S.C. §101 defines a U.S. Government work as a work prepared by a military service member or employee of the U.S. Government as part of that person’s official duties.

## Supporting Information

S1 Fig High-throughput GST expression and detection of *P*. *falciparum* proteins. Compartmental wheat germ expressions of all recombinant proteins are shown as GST fusions. [C14]-Leucine labeled proteins determined by autoradiographs in three fractions; total (T), supernatant (S), and pellet (P). The molecular sizes for GST-fused proteins (A) include an additional 29 kDa (GST molecular size).
(TIFF)

S1 Table Peptide pools and sequences of *P*. *falciparum* antigens tested by ELISpot described in Tables 2 and 3. Peptides were predicted as T cell epitopes for each subject HLA haplotype using predicative T cell epitope algorithms (see Methods). Each pool comprised ten 15mer peptides each containing a predicted epitope.
(DOCX)

S1 Text Irradiated sporozoite immunization of human subjects: Abstract. (DOCX)
