# Supplementary figures and images for: Discovery of Novel Plasmodium falciparum Pre-Erythrocytic Antigens for Vaccine Development
Source: PLoS One. 2015 Aug 20;10(8):e0136109. doi: 10.1371/journal.pone.0136109 (PMC4546230; doi:10.1371/journal.pone.0136109)

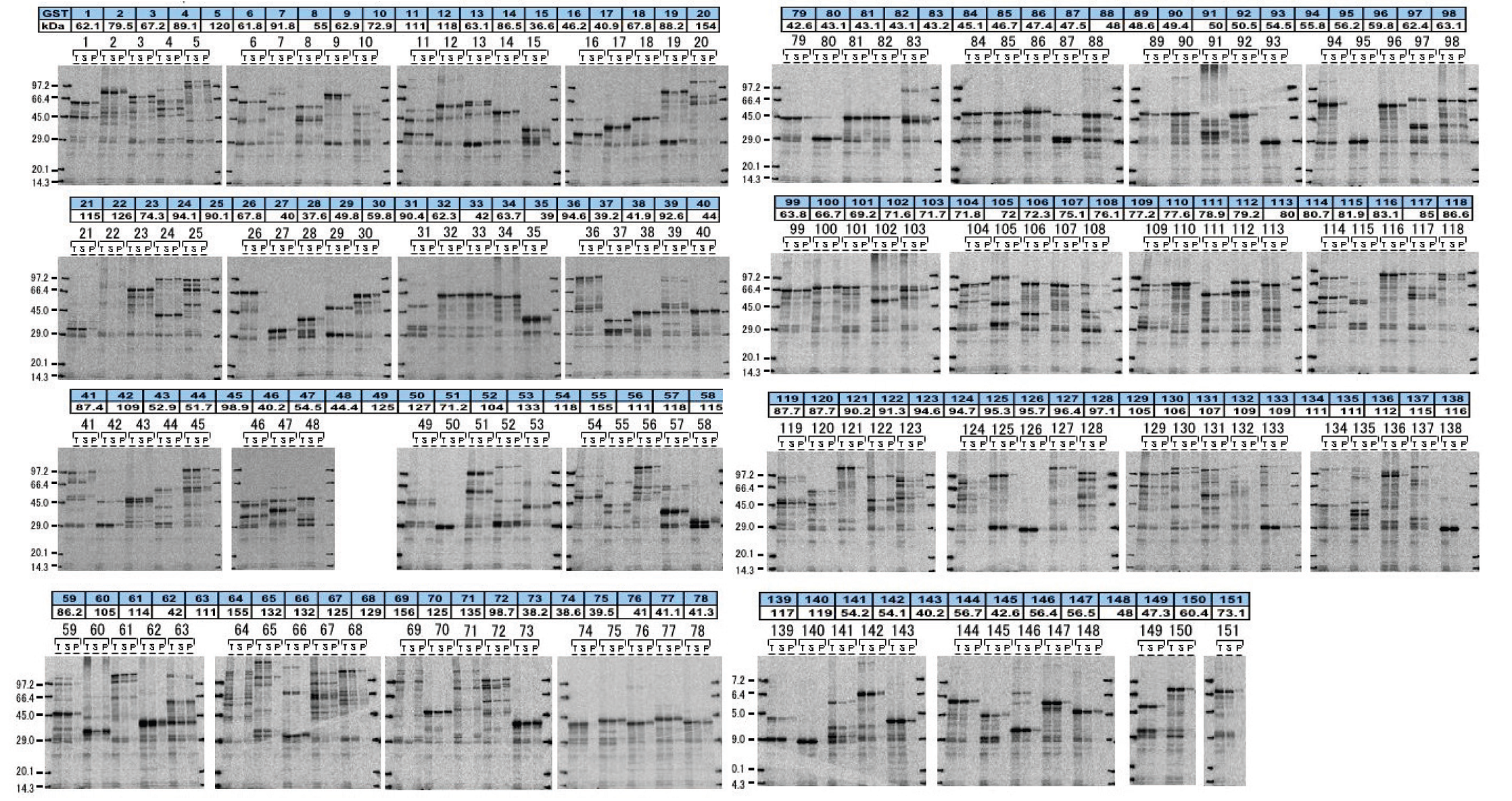

Supplement: S1 Fig — Compartmental wheat germ expressions of all recombinant proteins are shown as GST fusions. [C14]-Leucine labeled proteins determined by autoradiographs in three fractions; total (T), supernatant (S), and pellet (P). The molecular sizes for GST-fused proteins (A) include an additional 29 kDa (GST molecular size). (TIFF) [file pone.0136109.s001.tiff]
