# Supplementary material for: Discovery of Novel Plasmodium falciparum Pre-Erythrocytic Antigens for Vaccine Development
Source: PLoS One. 2015 Aug 20;10(8):e0136109. doi: 10.1371/journal.pone.0136109 (PMC4546230; doi:10.1371/journal.pone.0136109)
Supplement: S1 Table — Peptides were predicted as T cell epitopes for each subject HLA haplotype using predicative T cell epitope algorithms (see Methods). Each pool comprised ten 15mer peptides each containing a predicted epitope. (DOCX) [file pone.0136109.s002.docx]

**Table S1. Peptide pools and sequences of *P. falciparum* antigens tested by ELISpot described in Tables 2 and 3.**

| **Pf02 A02** | **Pf08 A02** | **Pf09 A02** | **Pf09 B07** |
| --- | --- | --- | --- |
| NQHLLGVLILPVLIL | YLELQDLLLKRFQVF | RLPHINFYLKFQKKD | STKNPRGYMKQMKLY |
| PVLILLSGELSVFVH | ILYEWAKFLIKENDN | SLFSLFSLFFIYLFI | KEIALRKHGSLLTVK |
| IFYCLMLVLSNYLLY | AHFLMLANLKYLELQ | IYLFIIIFVFPFVLK | IIFVFPFVLKNMIHS |
| GVLILPVLILLSGEL | DLLLKRFQVFKNEEI | KKNYNETLVHINIYL | MTTPFSLFSLFSLFF |
| TSLHTWQMWGCLITV | HSYVYAPCEMETSKE | KEIALRKHGSLLTVK | DYFDDRLPHINFYLK |
| PELLTAEILFVALTL | CEWLVNKIHISNDEQ | HLYLDIKDDIFHLDD | DFDTPVCINQNDNTY |
| NYLLYKTPCIASYPV | SNFYSLIKFPYQDIL | NSSSSTNKINKTSKL | TLTNPYDIHEIDNEY |
| FKGYLAILCVIIGRS | AKFLIKENDNTNITY | FVQNFFDKVTREQNA | LLTVKNLYNNKLLNI |
| CNVFVMYRLFLDVIP | KENDNTNITYIPQKV | INIYLCKECKALDNN | NNNNNNIHRDHLYLD |
| VALTLCNVFVMYRLF | YQDILNISKNIKNMS | KTSKLIHKFKKTKTI | CKECKALDNNFRMIS |
|  |  |  |  |
| **Pf26 B07** | **Pf56 A01** | **Pf56 B62 (B15)** | **Pf61 A02** |
| EYLNTPYYDYFDLYV | LVNNFSTKYVLIYEK | IATLSVGVQNTFQGP | YFCLFTILFWLSLVS |
| ETTDLAAILGSLDNS | NHTTATHTNNILYNP | LSYATFLFFSFILIN | YMAENKFNLPMSSEV |
| CNNDKVGDKNKKLSN | SEENELVNNFSTKYV | DIIKLLKDLIKYLHI | FLLEKISLITFVEDL |
| IIRFPFSVSKDIKKY | LKEMEESKLKMKYSK | EELLNEAYNAFSDYY | NYEKGLSLINLIVHS |
| HKTLDRNNLYKSNDV | NAKAGSLINHLSYAT | SLINHLSYATFLFFS | AKYLIFFFKNLHVWK |
| DIYFAEKLIYEYLNT | EAYNAFSDYYYFFPY | FKKLCNSKAAFNNTL | QIDKLNKTLFEIKNK |
| QPTTLLNSRFFLIHF | NIDEEGIRKLLENSF | PQTVILTVIQSFMLM | NHMMLSNEQFINKNK |
| PYYDYFDLYVKTLNE | FLITGILCELRNGNK | DIIKLLKDLIKYLHI | TILFWLSLVSGENVN |
| YKDLREVYFMNDNDL | TCVDDELKDIYKESI | KHYTKHGATSFIQSR | NIVLGKIHNILKDFN |
| NNNKQNEIVDETTDL | DDYLCSEPKYENICI | MIERLIKIHENLTKY | QNINYMNNYLNSIQY |
|  |  |  |  |
| **Pf61 A03** | **Pf78 B07** | **Pf84 A02** | **Pf116 A02** |
| KLNEVNDLKTIIIDE | QGEMKMAPPDYRIDI | EENLFEWSKTVTNHP | FGPKALPELPPQIYM |
| NMDNKKRMYNYNKHK | MLTVAGPRSQTELFE | MKLRILKKHYYVVFI | KVLPVCPVELLPKYP |
| TGDFIIKLYTNYVNY | QITFRPKVDIMYDAD | TNALKGYNIEVCEAG | SEFGPKPLPELPPQI |
| MNNYLNSIQYNKTLT | IFKLPNNILDDTAKA | VALDGAHGKVNRSKK | GPKPLPELPPRIETK |
| KVDVIDEKTKKKIAN | MAPPDYRIDIKEKPS | ESQLGGKITKIINVS | PLPELPPRIEMKLPK |
| GMMIKTDKKYLYNLK | PNTVLEIEPDNNLKK | EADSLIDPGYRAQIY | CPVELLPKYPTPTYP |
| NPKYMNYETYYKKIF | LVLHAKEREVGYFKR | PLDGTVLTGFKVEFH | FSKDSNIDYQPYVYT |
| TDEEKRILHISGVFK | DTAKATYKNGILEIK | STLQPVNGWIRKENA | KLVKQLPFEGESSYR |
| QNSELYPKDSKKFDK | EIEPDNNLKKQITFR | CENVSLWMKFFDIYG | PKPLPELPPKIYMQP |
| KLKDQIKITSDILYK | KEKPSIPKSKNALVN | KHTSVFPGLYFIGIG | KLVKPLPFEGESNYR |
|  |  |  |  |
| **Pf106 A02** | **Pf106 B07** | **Pf121 A03** | **Pf144 A02** |
| SCDQIIKLGDIINSV | YSYYRPFDPTRDTNT | NLLKKFHITKYDYKI | IGDTLSNKVGELWHS |
| VNNVLCINLDSVNGN | TTTNHFPKPREQLVG | NLLKKFHITKYDYKI | MNKFTYCITLLIALV |
| NFLSRYNNSVLNNMQ | FPKPREQLVGGSSML | KEYVLKTIYRKKNVD | HLNSFIDIVSTKVVN |
| FFCFFLLSCIVHLSR | KGNAPFNFTIIPYNY | KYNFNSSKRKIKK | LSNGNNLEKFMILLE |
| MNLLVFFCFFLLSCI | NPVDENNHIIDSTKK | EIQIENLLKKFHITK | IQKLEEQEKALLQ |
| NEKIISINSTVNNVL | IPYNYVNNSTEENNN | ESLKILKNAKHIKKN | KAKEKLLSLYQKHKG |
| EQLVGGSSMLISKIK | PHKPGKYFIVYSYYR | KDNLMVNRNIKYPSG | FSILLKSLVIPIAVQ |
| GNTKVPGNVPELKAR | NHQIPVSNIFSENID | EDEKVNINDAEKIDN | ATQKLDEQSKNIAQP |
| FNFTIIPYNYVNNST | PGNVPELKARIFSEE | VNTILPSWDIKYNFN | KKLGSKAKEKLLSLY |
| LISKNLKNSSNPVDE | ESAENNHTNSLNPNE | VNLAIKEYVLKTIYR | KLKDKFKNSKFGQKL |
|  |  |  |  |
| **Pf144 A03** | **Pf144 B07** | **Pf01 B07** | **Pf13 A02** |
| LALIQTEFIRKLKDK | PIAVQFIRKNLNKWK | DKNPPLLTNHILPNL | LKNHVLFLQMMNVNL |
| LLSLYQKHKGKLRHF | LIALVLPIRSIQLHS | NQPHPVLKPDGLLVG | ENILVDEFKKLKNHV |
| FKNSKFGQKLKKLGS | KSLVIPIAVQFIRKN | DNRPfEYQNNFPf | SVRLFEESLGIRKNK |
| IDIVSTKVVNKLEDD | KLRHFFSILLKSLVI | RILIHPEWRNNNHNY | MHDFFLKSKFNILSS |
| FGQKLKKLGSKAKEK | NIAQPIINRLYNSFE | MGPfGNEPNSDNRPf | DEFKKLKNHVLFLQM |
| IINRLYNSFEDKIEE | FDASAYEGKNLALIQ | LRYDPMGPfGNEPNS | MNVNLQKQLLTNHLI |
| LSNGNNLEKFMILLE | VNYAPLNYIEIGDTL | PNNKFFNPGKLRYDP | DKKKNGTMYILLKKI |
| IQLHSVNYAPLNYIE | LNKWKQRTLEATQKL | QIHTSDTYNLNILKI | YQNFQNADKNFLYLL |
| DELNALGQLDKDKQD | YCITLLIALVLPIRS | ITMKPHPQNCDKNYL | QKQLLTNHLINTPKI |
| LPIRSIQLHSVNYAP | QRTLEATQKLDEQSK | IEDPSSIHSCTINIQ | LLKKILSSRFNQMIF |
|  |  |  |  |
| **Pf13 B07** | **Pf51 B07** | **Pf51 B62 (B15)** | **Pf72 A01:03** |
| SVRLFEESLGIRKNK | FMVPMKFYINHEMYN | LLLEKTFYGSDTFRV | FIKECSDFNFNEYEQ |
| MPHHIIINNKTEVSS | EDNSVDVYLNNILVE | DVYLNNILVEYKYEN | HINYEYILKQGFIKY |
| LFLQMMNVNLQKQLL | KKRKSNATMAINRDK | TFYGSDTFRVFEDFD | EGEEITISYGNVNND |
| FKKDRNNNDDLGVMH | KKTPENIYKEIYENN | SLSHLKQYKNIYVNN | LLLLEYGFIEKEKET |
| TNHLINTPKIMPHHI | SYNLERKNNKYLNFL | IMKYTIIISGLVLLF | KTSNILRNYSTFFFY |
| MNNFYNNFNKNSLFN | EVEGEVKGKVVEGIE | NESYTFMVPMKFYIN | NVNNDLLLLEYGFIE |
| NQMIFVSSIFISFYL | NKTINTSYKNRVLKE | NLNHEKSYFHNKTIN | INELYNFIMLRFYEY |
| RMTSLKNELNEQLIY | DKHLPSYNLERKNNK | LVLLFCISFIIYYFD | KQLNIIDDNQSVYQR |
| EPTANLLQDKNKIND | FQENDNNDSVIMKYT | KSYFHNKTINTSYKN | YGFIEKEKETKVYFH |
| INKHWQRALKISQLQ | NIITRKKTPENIYKE | KKKSIDKHLPSYNLE | FDTKNLESAHITRKK |
|  |  |  |  |
| **Pf72 A02** | **Pf72 B07** | **Pf72 B62 (B15)** | **Pf77 A01** |
| LKYWDIKLTLVVTYI | INNAQVQIEPSLLKN | MLKHKYALYRCIRTI | ENVNKLIKYKIILKY |
| SKVNINHLTSWDHFL | VDKLPLIMLDSLEQV | VCTHAIKMKSPLNLY | IILVLLKCVLSYNLN |
| ILIKLIDEIIYEHFS | HYIQSTHPSALQNVK | SQNNHNQPLIPSSII | VDDFSDDIGFVKTSM |
| VDKLPLIMLDSLEQV | YALYRCIRTILISLL | HINYEYILKQGFIKY | LEEVSDHVVQNISKY |
| EINKLDSITILLYDI | YINILLYVYNFNNTL | INELYNFIMLRFYEY | SLTIEEKLNLFDGLL |
| HSNDLSSGHKNVNNN | LEPFKNVPKERNNIN | LQINMLHSHKSINLS | NNNFSLNTYVRKDDV |
| VVTYIHFISKYINIL | NQPLIPSSIIQNIQS | ILFTNNKIFIELDKI | EEGLSDDFKNNLSNY |
| YDFVMHIYSYVCTHA | CIRTILISLLIYIWL | IFNLFENIFNYDFVM | EIELVNFISTNYDKF |
| NYDNIIQEIQMFVNH | SVYQRFDTKNLESAH | YEHFSHINYEYILKQ | NIQKMVDDFSDDIGF |
| WDHFLSIDSEMVVIQ | FWERKLEPFKNVPKE | TIKYVAYNNSIFNLF | NDDIKEIELVNFIST |
|  |  |  |  |
| **Pf83 A02** | **Pf131 A02** |  |  |
| ILIFTIFSVVNIVVL | GYTEYEYLGLTLMNV |  |  |
| NIVVLLICVILSIKK | CEAQYVNEFLITVNY |  |  |
| MLMKISRYFFLLYLI | NFKLLELKNLLKKVK |  |  |
| FRTTYILIFTIFSVV | LLKILIGLCIKLGYK |  |  |
| KAHLDFFLRYRTGFI | NVEETLLKILIGLCI |  |  |
| NNRLLNEHAHCDAWS | SKILDEKYKSIMHQF |  |  |
| SRYFFLLYLIKAHLD | ELNLLFPDVCKIGKK |  |  |
| LLYLIKAHLDFFLRY | IYSRLLNESILLNKL |  |  |
| EKKKKFRTTYILIFT | QNNNMHSLKEQEQIV |  |  |
| FFLRYRTGFIRSRLE | NFKLLELKNLLKKVK |  |  |

Peptides were predicted as T cell epitopes for each subject HLA haplotype using predicative T cell epitope algorithms (see Methods). Each pool comprised ten 15mer peptides each containing a predicted epitope.
