# Supplementary material for: Discovery of Novel Plasmodium falciparum Pre-Erythrocytic Antigens for Vaccine Development
Source: PLoS One. 2015 Aug 20;10(8):e0136109. doi: 10.1371/journal.pone.0136109 (PMC4546230; doi:10.1371/journal.pone.0136109)
Supplement: S1 Text — (DOCX) [file pone.0136109.s003.docx]

**S1 Text. Irradiated sporozoite immunization of human subjects: Abstract**

B. W. Hickey, J. M. Lumsden, S. Reyes, Y. Elbeshbishi, A. Mix, J. Spurgeon, T. Luke, D. Freilich, E. Villasante, J. Epstein and T. L. Richie. 2013. A Retrospective Analysis of Adverse Event Data from the Phase 1 trial *Plasmodium* Sp Sporozoites Immunization of Human Volunteers. Am Soc Trop Med Hyg. Annual Meeting, Abstract 1161.

In this clinical trial, healthy adult research subjects were immunized with radiation-attenuated *Plasmodium falciparum* sporozoites (*Pf*RAS) by mosquito bite, a model for immunization against malaria that induces high grade (>90%) sterile protection against controlled human malaria infection (CHMI). Leukapheresis was conducted before and after *Pf*RAS immunization to collect large numbers of peripheral blood mononuclear cells (PBMC) to characterize protective immune responses and identify protective antigens to support malaria vaccine development. Subjects were true-immunized with *Pf*RAS or mock-immunized with uninfected mosquitoes. We here present the safety, tolerability, protective efficacy, and humoral response data from the trial.

Fifty-seven subjects were screened, 41 enrolled, and 30 received at least one immunization. Local (site of mosquito bite) adverse events consisted of erythema, papules, swelling, and induration; one true and one mock immunized subject developed generalized swelling of the forearm (large local reactions) and were withdrawn from further participation. Systemic AEs were generally rare and mild, consisting of headache, myalgias, nausea, and low grade fever; however, two true-immunized subjects were awaken from sleep 16 hours after immunization experiencing acute symptoms including fever, malaise, myalgia, nausea and rigors. These reactions, consistent with serum sickness, may have resulted from pre-formed antibody reacting with mosquito salivary antigens. Ten of the subjects immunized with *Pf*RAS underwent CHMI by mosquito bite and five (50%) were protected against malaria infection. All developed humoral immune responses to whole sporozoites and to the circumsporozoite protein (CSP) prior to CHMI. Mean sporozoite IFA titers and CSP ELISA titers were higher in protected than non-protected groups although the differences were not statistically significance.

Overall, immunization with *Pf*RAS via mosquito bite was safe and well tolerated in most research subjects.  Large local reactions and severe systemic adverse reactions occurred in four individuals, likely representing allergic responses or serum sickness following injection of mosquito salivary gland antigens.
